# Revising Substantial Leg Length Discrepancy in Total Hip Arthroplasty Using Computer-assisted Navigated Systems: A Case Series of Three Patients

**DOI:** 10.7759/cureus.5137

**Published:** 2019-07-15

**Authors:** John M Dundon, Rachel R Mays

**Affiliations:** 1 Orthopedic Surgery, Orthopedic Institute of New Jersey, Morristown, USA; 2 Clinical Research, Intellijoint Surgical, Waterloo, CAN

**Keywords:** total hip arthroplasty, leg length discrepancy, intraoperative navigation, computer-assisted navigation system, patient navigation, intra-operative navigation

## Abstract

One of the primary challenges of total hip arthroplasty (THA) is equalizing the limb lengths to re-establishing normal hip biomechanics. Post-operative leg length discrepancies (LLD) lead to patient dissatisfaction and are a main source of orthopedic malpractice cases. The aim of this case series was to document three cases of substantial LLD that were corrected during THA with the assistance of an imageless computer navigation system. Medical records were reviewed for history and radiographs were consulted. All patients in this series presented with complaints related to hip fractures and reported a significant lengthening of leg length following THA. No surgical complications of adverse events were reported. In these cases, imageless navigation provided intraoperative measurements of leg length which allowed for enhanced accuracy of component placement and improved outcomes following surgery.

## Introduction

Total hip arthroplasty (THA) is among the most efficacious procedures in current orthopaedic practice. One of the primary goals of THA is equalizing the limb lengths to re-establish normal gait biomechanics. Restoring equal leg lengths can help facilitate a return to regular gait and function as well as relieve pain [1]. The incidence of post-operative leg length discrepancies (LLD) has been reported to range anywhere from 1 to 27% and LLD is indicated to vary from 3 to 70 mm [2, 3]. The Joint Commission on Accreditation on Healthcare organizations reported that LLD after THA were major adverse events, leading to 4.7% of medical errors [4]. Post-operative LLD has also been associated with back pain and sciatica, neuritis, gait disorders, dislocation, pre-mature loosening of components as well as patient dissatisfaction [5-7]. As a result of these negative patient outcomes, LLD is one of the most common reasons for litigation after THA [8]. With the demand for primary THA anticipated to double by the year 2026, orthopedic surgeons must continue to find avenues in which they can improve their surgical accuracy and techniques in order to reduce such complications [9].

Current intraoperative manual methods used to monitor leg length changes are erroneous and rely heavily on surgeon experience. These techniques, such as mechanical pins, osteotomy or tissue tensioning, lack the ability to accurately measure patient position during surgery. Computer-assisted navigation is an increasingly common addition to THA surgery and has proven the ability to improve the precision of leg lengthening [10]. Traditional computer-assisted surgery does improve accuracy and help reduce the likelihood of significant LLD, yet the devices are expensive and disrupt the normal workflow of the performing surgeon. Recently, imageless computer navigation systems have been introduced as a possible improvement on traditional image-based navigation systems. These devices are smaller in size and more sophisticated, with minimal alteration to the surgical workflow, which enables faster judgments and diminishes the potential for costly LLDs. Here, we report three cases of substantial LLD preceding THA, performed with the assistance of an imageless computer navigation system.

## Case presentation

Case 1

A 78-year-old male presented with chief complaint of chronic and worsening left hip pain. Relevant history included an intertrochanteric hip fracture that had been treated one year prior with a cephalomedullary nail. Prior to hip fracture, the patient could walk without any assistive devices and was a high-functioning community ambulatory. Following the injury, the patient was limited to ambulation with the assistance of a walker. On physical examination, the left leg was significantly shorter than the right leg. Range of motion revealed an extremely limited range of the hip and impingement testing was positive. Infectious workup was negative for an infectious source. Neurological testing was unremarkable. Radiographs of the pelvis were obtained and demonstrated a hip fracture with a non-union of the intertrochanteric region of the femur, as well as penetration of the head screw through the femoral head. There was also screw penetration into the acetabulum and an LLD radiographically measured at 45 mm (Table 1).

**Table 1:** Demographic, relevant history and complications of all included cases pre-THA. THA: Total hip arthroplasty

Case	Age, laterality, gender	Relevant history	Complications
1	78, left hip, male	One year following intertrochanteric hip fracture that was treated with a cephalomedullary nail.	-failed, non-union of the intertrochanteric fracture, and -penetration of the head screw through the femoral head and the acetabulum.
2	63, right hip, female	Six months following a fall and displaced femoral neck fracture; had cemented right hip hemiarthroplasty.	-failed, displaced femoral neck fracture, -sciatic nerve injury, and -foot drop (heterotopic ossification).
3	76, right hip, female	Four months following intertrochanteric hip fracture.	-failed, non-union of the femoral neck, and -penetration of the head screw through the femoral head and the acetabulum.

**Figure 1:**
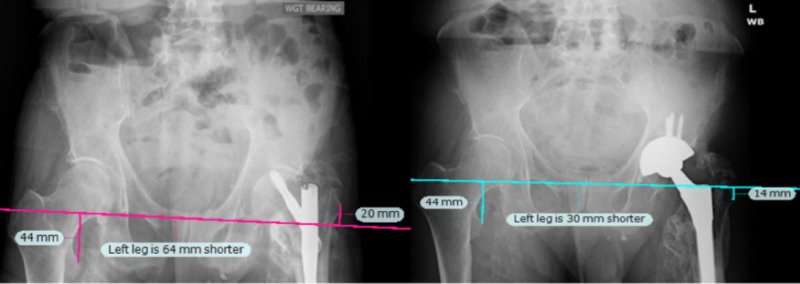
Radiographs for Case 1. Due to deformity at the operative lesser trochanter, the greater trochanter was used as a landmark for leg length calculations. Preoperatively (left), the LLD was estimated at 45 mm. The operative leg was lengthened by 35 mm during surgery, which was confirmed by both the navigation device and the post-operative radiograph (right). LLD: Leg length discrepancy

The preoperative plan included left hip THA. During surgery, computer-assisted navigation was used to assist with component placement and monitoring of changes in leg length. Intraoperative navigation measurements indicated a lengthening of 35 mm, which was confirmed on post-operative radiographs. Surgery was successful and the patient progressed well, experiencing significant pain relief and satisfaction with his surgery. He was discharged to home with a walker and within six weeks of follow-up, the patient had transitioned to walking with only a cane. At three months the patient was no longer using any assistive walking devices and at one year follow-up the patient was pain free and returned to his previous level of function prior to the hip fracture. Radiographs revealed that the left hip was well-aligned, with the left leg lengthened by 35 mm for a significantly improved post-operative LLD of 10 mm (Table 2, Figure 1).

**Table 2:** Pre- and post-operative leg lengths of all included cases, measurements were reported by device. LLD: Leg length discrepancy

Case	Pre-LLD (mm)	Post-LLD (mm)	Lengthened (mm)
1	45	10	35
2	37	2	35
3	30	0	30

Case 2

A 63-year-old female presented with a chief complaint of right hip pain. She reported a fall six months previously, which resulted in a displaced femoral neck fracture and was treated surgically with a cemented hemiarthroplasty. She described no relief from this procedure and had pursued relief through other conservative treatments without success. On physical examination, the right leg was substantially shorter, with a distinguishable LLD present. Neurological testing revealed that the index surgery was complicated by a sciatic nerve injury and the patient had a foot drop on the operative side. This had developed into significant heterotopic ossification. Radiographic examination of the pelvis revealed a substantial LLD of 37 mm. Infectious workup on the right hip was negative.

**Figure 2:**
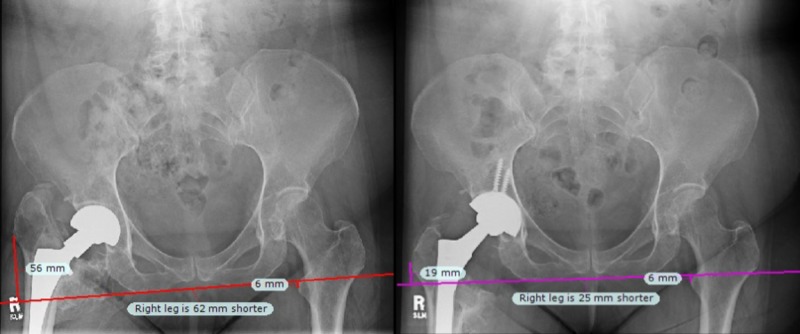
Radiographs for Case 2. Due to deformity at the operative lesser trochanter, the greater trochanter was used as a landmark for leg length calculations. Preoperatively (left), the LLD was estimated at 37 mm. Post-operatively (right), the operative leg was lengthened by 35 mm, which was confirmed by the navigation system and radiographs. LLD: Leg length discrepancy

Following consultation with the patient, the preoperative plan included a revision of the hemiarthroplasty and conversion to a right hip THA. During surgery, computer-assisted navigation was again used to assist with component placement and monitoring of changes in leg length. Surgery was successful and the patient did well post-operatively. She reported significant pain relief and was satisfied with the outcome of her surgery. Radiographs revealed equalized leg lengths and implants were well aligned. The patient was able to ambulate by three months post-procedure without any assistive devices. Her foot drop from the index surgery remained stable and improved slightly over the next year. A leg lengthening of 35 mm was confirmed on post-operative radiographs, resulting in a post-operative LLD of 2 mm. At one year the patient was back to her normal functional status prior to her hip fracture (Figure 2).

Case 3

A 76-year-old female presented with right hip pain. Relevant history included a prior intertrochanteric hip fracture treated with a cephalomedullary nail with subsequent non-union and failure, occurring four months previously. Prior to her first hip fracture the patient was able to ambulate without any assistive devices and was a high functioning. Following hip fracture, the patient was limited to minimal ambulation with a walker and used a wheelchair for distances. On physical examination, the right leg was significantly shorter, with the LLD discrepancy measured grossly at approximately 30 mm. Range of motion was extremely limited and impingement testing was positive. Infectious workup was negative for an infectious source. Neurological examination was unremarkable. Radiographs revealed the patient had penetration of the head screw through the femoral head and penetrating the acetabulum. The radiographs also showed that the LLD was at 30 mm preoperatively.

**Figure 3:**
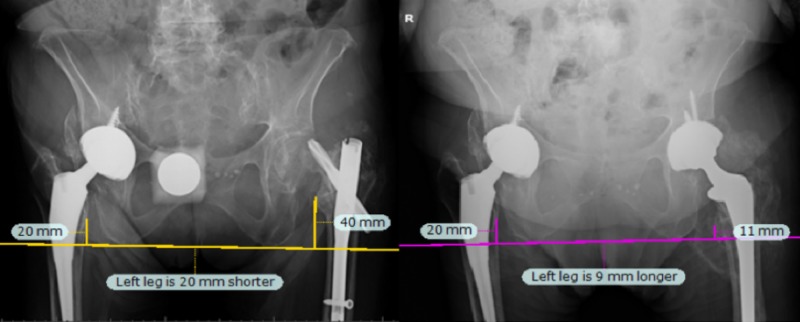
Radiographs for Case 3. Preoperatively (left), the LLD was estimated at 30 mm. During surgery, the operative leg was lengthened by 30 mm, giving equal leg lengths, which was confirmed by navigation and post-operative radiographs (right). LLD: Leg length discrepancy

Following consultation with the patient, the perioperative plan consisted of a conversion of the previous surgery and intertrochanteric mal-union to a THA, with soft tissue release and removal of the previous heterotopic bone. An imageless navigation system was used in order to help restore leg length and set the cup abduction and version given the large leg length discrepancy, massive heterotopic ossification, and destruction of the native acetabular anatomy. Surgery was successful and the patient was discharged to a rehabilitation facility with the assistance of only a walker. She described meaningful pain relief and was content with the outcome of her surgery. Within six weeks of follow-up, the patient had transitioned to a walker from the wheelchair and at three months follow-up the patient was walking without pain in the right hip. The right leg was lengthened by 30 mm, resulting in equalized leg lengths and well-aligned hips (Figure 3).

## Discussion

Here we report three cases of substantial leg length discrepancies corrected with the assistance of computer-assisted navigation technology. Leg length equality following THA can have a meaningful impact post-surgery on both the quality of life and functional outcomes for the patient. Furthermore, significant LLD can lead to additional revision surgeries as well as an increased possibility of litigation.

Discrepancy of leg length is common after THA and consequently so is patient dissatisfaction. Approximately 30% of patients can perceive a change in LLD post-THA [11]. Edeen et al. reported that even a minor discrepancy could induce patient dissatisfaction; yet, the actual clinically acceptable amount of LLD has not yet been fully established [12,13]. One study reported that LLD can be perceived when shortening exceeds 10 mm and lengthening 6 mm [14]. Likewise, an excess of 10 mm of lengthening after THA considerably impairs positive clinical outcome, resulting in a 27% and 18% reduction of mean Oxford Hip Score (OHS) at three and 12 months, respectively [15]. Patients with even a modest amount of LLD report discomfort for the first few months that declines with time, although 15% remain symptomatic and need a compensatory shoe lift to account for the differences [13]. Lengthening more than 10 mm, reported in 16 to 32% of THA patients, has been associated with limping, pelvic obliquity, a need for a shoe lift plus a feeling of dissatisfaction [16].

The cases presented in this report utilized an imageless, computer-assisted navigation system (Intellijoint HIP®, Intellijoint Surgical Inc., Waterloo, ON) to assist with component placement. This device provides data that is integral in equalizing leg lengths during surgery, such as real-time intraoperative data concerning acetabular cup position and changes in leg length. This ability to closely monitor changes in leg length during trialing is paramount in managing leg length changes, especially in challenging cases where lengthening is significant. This is especially prevalent in cases such as those presented in this report, where injury or deformity results in a significant leg length inequality, or in cases of dysplasia or other developmental conditions. Indeed, the device we utilized has been used to successfully monitor significant changes in leg length in cases of Legg-Calve-Perthes disease, where in two cases, preoperative radiographs revealed LLDs of 25 mm and 35 mm [17]. In each case, the navigation device provided important intraoperative data resulting in equalized post-operative leg lengths, which was confirmed by post-operative radiographs. This translates into better stability, performance and patient survivorship.

While maximizing improvements in patient-related outcomes is key to success in orthopedic procedures, post-operative LLDs have other substantial impacts, including economic. Orthopedic surgery trails only obstetrics and general surgery as the most high-malpractice risk surgical speciality, typically due to the risk of these post-surgical complications [18]. For orthopedic surgeons, post-operative leg length inequalities rank among the most common causes of litigation, with a recent statistic indicating that the average insurer payout after complications following primary or revision THA was, per claim, approximately $73,457 per surgeon [19]. Poor leg length management during THA represents a potentially costly consequence to clinicians as well as the hospitals participating in surgeries. The use of navigation technologies may play a role in improving these outcomes by impacting the potential cost-effectiveness of the episode of care. Recent evidence indicates that computer-assisted surgery may be more cost-effective than manual surgery [20]. As such, third-party payers may see value in increasing their reimbursement to hospitals and surgeons that employ this technology, if it can be further proven to reduce complications and prevent costly revisions. More research is required in this area.

## Conclusions

This report summarizes three cases of significant change in leg length following THA with the assistance of an imageless, computer-assisted navigation device. The device provided intraoperative measurements of leg length which allowed for improved accuracy with component placement and improved outcomes following surgery.
